# The Lighting of Operating Theatres

**Published:** 1917-10-13

**Authors:** 


### THE LIGHTING OF OPERATING THEATRES.

### By Day and by Night.

The provision of adequate natural lighting of all rooms used for both minor and major operations is a point of the first importance, and one which is fully recognised by all architects specialising in hospital construction. A north aspect for the main windows is selected, and the area should be the very maximum attainable, and starting from, say, three to four feet above the floor line. The lower half or three-quarters of the panes are usually frosted, which has a dispersive effect on the light and assists to reduce hard shadows. As the windows are not as a rule made to open, the panes are made as large as practicable. The white walls and ceilings, of course, materially assist in neutralising shadows, and for the smaller rooms the lighting effect is generally ample. For the larger theatres a top light also is usually provided where the site permits.

In many of the less modern buildings the natural light has to be supplemented by artificial means, and in every case this should be available both day and night, and in fact some surgeons make a practice of working with the electric light turned on in unfavourable or overcast weather in case of the daylight suddenly failing.

As regards the artificial light to be provided, the general lighting presents no difficulties, and a few wall brackets, with fairly opaque shades to shield the lamps, usually suffice. Electric lighting is, of course, invariably installed where available. The fittings should be of the simplest design and preferably made watertight, so that the walls can be washed down.

The artificial illumination of the table is a much more difficult problem, and several methods are now in vogue; the latest system for the larger theatres provided with top daylight embodies the use of very high-powered lights arranged above the glass skylight, and provided with reflectors, the idea being as far as possible to reproduce daylight effect. This system is claimed as being the most efficient as it is certainly the most expensive in use to-day, and has the advantage that none of the apparatus is actually in the theatre.

### A Perfected Light.

Another method makes use of a very powerful light, usually an arc light, fitted in an adjoining room and reflected by a system of mirrors on to the desired position. In any case it is better for the actual operating light to be supplied, if possible, from an entirely separate source, in case of failure of the general lighting. In some important hospitals storage batteries are installed for this sole purpose where local lights are in use over the tables.

For the lighting of the operating tables in the smaller hospitals, the adoption of either of these schemes is, of course, generally out of the question on the score of the expense not being justified for the limited use of the rooms at night-time, and some form of local lighting apparatus has to be used. Up to a short time back there was nothing on the market which was entirely satisfactory. What is required is a light which can be adjusted to any angle, position, or height, and remain in any required position without attention, and at the same time be capable of being removed out of the way in the day-time. The apparatus should also be designed so that the surgeons should have free access to the table from all sides.

To meet these requirements in the smaller operating rooms at the new King's College Hospital, Professor Capper, the consulting engineer, who was responsible for the electrical equipment there, perfected a new type of fixture, after considerable experimenting and consultation with many leading surgeons, which not only fulfils these conditions, but possesses the advantage that the position of the light can be adjusted from time to time, if required, without the necessity of approaching the table, as the attendants can do this from the position on the wall where the apparatus is attached.

The actual source of light is a bunch of incandescent lamps contained in a special form of aluminium reflector, giving a fairly wide distribution of light and at the same time amply powerful. The reflector is attached by means of a light but rigid bracket to raising and lowering and swivelling gear which is fixed to the wall. The whole thing is extremely neat and simple, and although the bracket is made as long as eight or nine feet it can be operated by a nurse with the greatest ease.

